# HIV prevention is not enough: child survival in the context of prevention of mother to child HIV transmission

**DOI:** 10.1186/1758-2652-12-36

**Published:** 2009-12-11

**Authors:** Louise Kuhn, Moses Sinkala, Don M Thea, Chipepo Kankasa, Grace M Aldrovandi

**Affiliations:** 1 Gertrude H. Sergievsky Center, and Department of Epidemiology, Mailman School of Public Health, Columbia University, New York, NY, USA; 2 Catholic Medical Mission, Lusaka, Zambia; 3 Center for International Health & Development, Boston University School of Public Health, Boston, MA, USA; 4 University Teaching Hospital, University of Zambia, Lusaka, Zambia; 5 Children's Hospital Los Angeles, University of Southern California, Los Angeles, CA, USA

## Abstract

Clinical and epidemiologic research has identified increasingly effective interventions to reduce mother to child HIV transmission in resource-limited settings These scientific breakthroughs have been implemented in some programmes, although much remains to be done to improve coverage and quality of these programmes. But prevention of HIV transmission is not enough. It is necessary also to consider ways to improve maternal health and protect child survival.

A win-win approach is to ensure that all pregnant and lactating women with CD4 counts of <350 cells/mm^3 ^have access to antiretroviral therapy. On its own, this approach will substantially improve maternal health and markedly reduce mother to child HIV transmission during pregnancy and delivery and through breastfeeding. This approach can be combined with additional interventions for women with higher CD4 counts, either extended prophylaxis to infants or extended regimens of antiretroviral drugs to women, to reduce transmission even further.

Attempts to encourage women to abstain from all breastfeeding or to shorten the optimal duration of breastfeeding have led to increases in mortality among both uninfected and infected children. A better approach is to support breastfeeding while strengthening programmes to provide antiretroviral therapy for pregnant and lactating women who need it and offering antiretroviral drug interventions through the duration of breastfeeding. This will lead to reduced HIV transmission and will protect the health of women without compromising the health and well-being of infants and young children.

## Introduction

The field of prevention of mother to child HIV transmission (PMTCT) has given us some of the biggest breakthroughs and achievements in the field of HIV prevention research. Notably, clinical and epidemiologic research in this field established that antiretroviral drugs are effective agents of HIV prevention. This field has also given us some the most heartbreaking disappointments and missed opportunities for resource-poor areas. Prominent among these are the increased child mortality among both infected and uninfected infants that has resulted from encouraging HIV-infected women to shorten the duration or abstain from all breastfeeding. Missed opportunities include the failures to implement widely effective antiretroviral and counselling interventions. Recent findings provide important new evidence to policymakers in crafting sound programmes for PMTCT and infant feeding.

Prevention of HIV transmission is not enough. It is necessary also to consider ways to improve maternal health and protect child survival. In this report, we review the available data and conclude that the key to future success in most resource-limited settings is to provide counselling and support for breastfeeding while strengthening programmes that provide antiretroviral therapy for pregnant and lactating women who need it and offering antiretroviral drug interventions throughout the duration of breastfeeding. This will lead to reduced HIV transmission and will protect the health of women without compromising the health and well-being of infants and young children.

## Discussion

### Discovery that antiretroviral drugs are effective agents of HIV prevention

The field of PMTCT can be credited with what is perhaps the biggest breakthrough in HIV prevention research. Fifteen years ago, clinician-scientists in the US and France demonstrated that zidovudine alone could reduce mother to child transmission of HIV from 25% to 8% in a non-breastfeeding population [1]. The use of antiretroviral drugs to prevent HIV transmission was controversial at the time, but the results were compelling. However, it was not clear how the complex and expensive regimen tested in the first trial could be made applicable to the health care circumstances in sub-Saharan Africa, where it was needed most.

Through the dedication and determination of investigators in the field, the successes of PMTCT research continued. Through well-coordinated and collaborative trials, breakthroughs continued, and studies in west Africa and Thailand demonstrated that short courses of oral zidovudine were effective in reducing transmission [2,3]. A landmark study in Uganda demonstrated that single-dose nevirapine was effective in reducing mother to child HIV transmission [4]. These two findings provided the first proof that feasible and effective antiretroviral prophylaxis could be implemented in developing areas, where health system resources were highly limited.

Active research in this area produced continued refinements in prophylactic antiretroviral drug regimens. Studies in Malawi and South Africa demonstrated how post-partum prophylaxis could be effective for the large number of infants whose mothers had not accessed optimal antenatal care [5,6]. A pivotal study in Thailand was particularly useful in demonstrating the improved outcomes with combination zidovudine and nevirapine prophylaxis [7]. With the remarkable increases in access to effective HIV treatment among adults in many African countries, studies began to clarify how best to integrate adult treatment and PMTCT activities [8]. Recently, two important studies demonstrated that using extended infant prophylaxis could reduce transmission through breastfeeding [9,10].

### Opportunities to implement PMTCT taken and missed

The demonstration that effective PMTCT is achievable has inspired civil society. Demands for programmes to provide access to antiretroviral drugs to prevent transmission to infants mobilized community organizations and the medical community in South Africa in 2001, resulting in the establishment of a national PMTCT programme. But investigators in this field have also been targets of attack and have borne the brunt of unfair accusations about poisoning babies and neglecting women. It has taken great courage to move these results into programmes.

In part through strong support from the international community, PMTCT has now been implemented in almost every African country [11]. There have been several sites with creative methods to adapt the interventions to be appropriate to the specific health service needs [12]. For example, in rural Uganda, a special pre-packaged formulation of the infant nevirapine dose was used to ensure that women who delivered at home could give their infants the nevirapine [13]. In Zambia, establishment of rigorous data management systems and careful analysis of routine data has allowed on-going strengthening of programmes to improve coverage and quality [14,15]. Implementation of PMTCT also helped leverage resources for HIV treatment programmes and has provided a platform to help treatment programmes get started.

Despite these encouraging successes, major gaps in PMTCT implementation exist. We are a long way from widespread implementation of interventions shown to be effective in clinical trials. Equitable and universal coverage is lacking in most countries. The global average is estimated to be that about 45% of women who need PMTCT have access to it [16], and this is highly skewed towards women living in certain urban areas. There is weak or non-existent coordination between PMTCT and adult treatment programmes in many places, and both continuity of care and follow up is very often poor. Quality of counselling, particularly as it relates to infant feeding, is of poor quality and often confused [17].

So, despite successful and clinically relevant PMTCT research, the human toll of infection among women of child-bearing age and among children remains high. The human toll is mostly borne by the young mothers with HIV who have to grapple with the terrible fear of transmitting HIV to their infants. A mother living in Soweto participating in an early infant diagnosis programme typifies the dilemma: "I told myself that just like in soccer ... I should prepare myself to either win or lose, positive or negative." [18] This mother teaches us that despite the formidable opponent of HIV, we should not give up hope.

### Making sense of the numbers: denominators matter

In the absence of any interventions, about a third of infants born to HIV-infected mothers acquire HIV infection. About 20% of infants born to HIV-infected mothers acquire HIV during pregnancy or delivery, usually referred to intrauterine and intrapartum transmission (or together as perinatal transmission) [19]. Approximately 14% of infants who are breastfed for the typical duration of 18 months will acquire HIV through breastfeeding [20]. These rates, which use the number of infants born to HIV-infected mothers as the denominator, have been known since the late 1980s and have been confirmed with more sophisticated study designs and with the latest HIV diagnostic methods [21,22].

**Figure 1:**
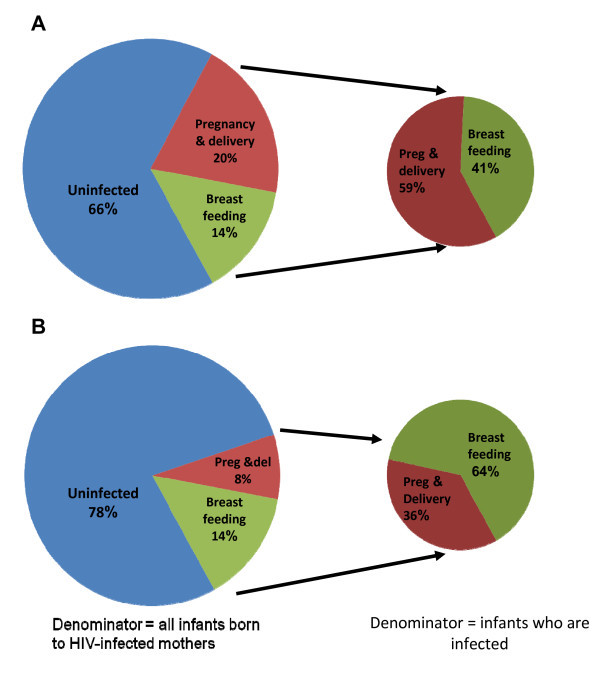
**Transmission rates and proportions of infections**. Panel A - Transmission rates and proportions of infections due to transmission through different routes among breastfed infants born to HIV-infected mothers if no interventions are provided. Panel B - Transmission rates and proportions of infections among breastfed infants born to HIV-infected mothers when short course antiretroviral interventions are provided.

If we re-express these rates using the number of infected children as the denominator, breastfeeding accounts for about 40% of all infant infections (Figure 1A). Careful epidemiologic studies over many years have identified several risk factors for transmission, the strongest of which are maternal CD4 count and viral load. Additionally, it has now been firmly established that breast milk transmission is substantially enhanced by the amount of virus in breast milk, and the quality and duration of breastfeeding [23,24]. Susceptibility factors, including innate and acquired immune responses in the child, in the mother and in breast milk, protect against HIV transmission [25,26].

Antiretroviral interventions given only during pregnancy and peri-partum reduce transmission occurring during pregnancy and delivery, and largely leave postnatal transmission untouched. While there may be some short-term benefits for early postnatal HIV transmission of some peri-partum antiretroviral drug interventions [27], they have only minor impact on transmission occurring throughout the full duration of breastfeeding.

For the purpose of illustration, consider the implementation of antiretroviral intervention that reduces intrauterine and intrapartum transmission to 8%. Assuming breastfeeding transmission risk is unaffected, an additional 14% acquire infection postnatally, bringing the total transmission rate to 22%. In this scenario, postnatal transmission now constitutes 64% of all infections, even though the total number of infections has shrunk. Many highlight the 64% statistic to emphasize the importance of postnatal transmission. But it is easy to misinterpret these numbers because of shifting denominators.

Transmission rates are expressed as the number of infected infants divided by the number of infants born to HIV-infected mothers. However, it is important to emphasize that the 64% statistic includes a *different*, more limited denominator - only infected infants born to HIV-infected mothers (Figure 1B). This distinction becomes essential to understand for health education, where messages about the actual magnitude of postnatal HIV transmission may become exaggerated.

### Breakthroughs in preventing HIV transmission through breastfeeding

It is an unfortunate reality that breakthroughs in confirming interventions to reduce HIV transmission through breastfeeding have been made only relatively recently. As result, the 14% or 64% *[sic] *postnatal HIV transmission rates remain unmodified in most education and training materials for PMTCT programmes. Thus the information that is provided is out of date and fuels the fear of women and counsellors (and often policy makers) about breastfeeding.

There have, however, been three crucial breakthroughs in understanding how to prevent postnatal HIV transmission that now confirm that it is possible to reduce postnatal HIV transmission to ~1%. These effective interventions include: (1) lactation counselling and support; (2) use of triple antiretroviral drug regimens for women that are either continued life-long as therapy or continued through breastfeeding as prophylaxis; and (3) extended regimens of antiretroviral prophylaxis to the infant.

#### Lactation counselling and support

The new millennium brought remarkable new insights from the Durban group, demonstrating that the quality of breastfeeding practices affects postnatal HIV transmission [24,28]. It showed that the risk of postnatal HIV transmission was reduced among women who breastfed their infants only breast milk and nothing else (exclusive breastfeeding) to three months, and was higher among women who introduced other foods and liquids earlier than three months while continuing to breastfeed [24,28]. The benefits of exclusive breastfeeding in reducing postnatal transmission have subsequently been confirmed, and refined, in at least four other large studies [29-32]. The biological basis of this association remains unknown and is likely to be multi-factorial [33].

The powerful insight provided by these observations is that a simple behavioural intervention of improving breastfeeding quality is as effective in reducing HIV transmission as the short-course antiretroviral drug regimens used for PMTCT. This is not to say that counselling should replace antiretroviral interventions. Rather, they are complementary interventions that should be implemented together. The benefits of lactation management and support extend beyond reductions in HIV transmission. Exclusive breastfeeding is best for all infants, regardless of their HIV status, and is associated with decreased infant mortality [34]. Another advantage of lactation counselling is that simple and consistent infant feeding messages can be given to both HIV-positive and HIV-negative women.

#### Therapeutic antiretroviral regimens for the mother

Treating pregnant women with combination antiretroviral drugs is highly effective in reducing vertical HIV transmission, including transmission through breastfeeding [35-39]. There are a number of ways in which antiretroviral therapy can be used. Standard treatment regimens can simply be given to all HIV-infected pregnant women, regardless of their clinical stage or CD4 count. This is an attractive approach for its simplicity, and it has been shown to be effective [35-39].

Another approach is to optimize the regimens to avoid toxicity and drug-resistance problems. Important new results showing the benefit of this approach from Botswana were recently presented [40]. A highly attractive approach is one based on rational integration of adult HIV treatment with PMTCT programmes [41]. In this approach, HIV treatment is provided only to women who need it for their own health. The effectiveness of this approach was demonstrated in the MTCT-Plus programme in Cote d'Ivoire [8]. This is a win-win intervention that treats the mother while preventing transmission.

It is important to understand why rational integration of adult HIV treatment programmes and PMTCT is so effective to reduce postnatal transmission through breastfeeding. It is clearly established that CD4 counts are a very strong predictor of postnatal transmission: the lower the CD4 count, the higher the transmission. In our study in Zambia [42], the postnatal HIV transmission rate through breastfeeding was 20% in women with CD4 counts of <350 cells/mm^3 ^and around 4% in women with CD4 counts above 350.

**Figure 2:**
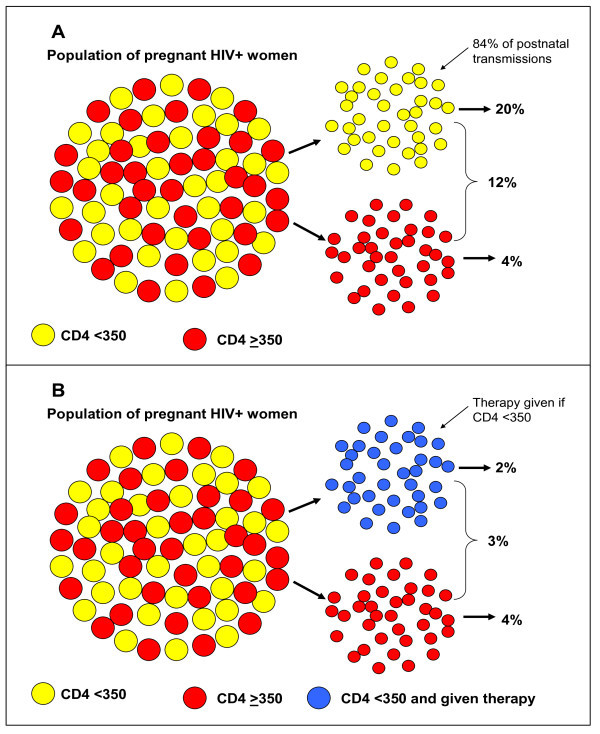
**Why treating only women with low CD4 counts reduces postnatal HIV transmission**. Panel A - Postnatal HIV transmission rates in the population are an average of low rates among women with high CD4 counts and high rates among women with low CD4 counts. Panel B - When antiretroviral therapy is given to women with low CD4 counts, the postnatal HIV transmission rate in this group, and in the overall population, declines to low levels.

Combining these two populations together yields the estimate of ~12%, usually quoted as the risk of postnatal HIV transmission through breastfeeding. Marked differences in the distribution of CD4 counts across study populations also explain why studies utilizing similar interventions may report such different transmission rates. With the distribution of CD4 count observed in our cohort in Zambia, more than 80% of all postnatal infections occurred among women with CD4 counts of <350 (Figure 2A).

If antiretroviral treatment is provided to all pregnant women with CD4 counts below 350, the available data indicate that postnatal transmission will be reduced to 1-2%. Even if no additional postnatal interventions are provided for women with higher CD4 counts, the transmission rate in the population as a whole will still decline to <5% (Figure 2B).

#### Extended regimens of antiretroviral prophylaxis to the infant

It is possible to reduce the transmission rate even further by providing additional interventions for those women with higher CD4 counts who do not need antiretroviral therapy for their own health. Two new studies from Malawi have shown that extended prophylaxis with nevirapine to the infant substantially reduce postnatal transmission through breastfeeding. In the first study, prophylaxis was used for only 14 weeks [10]. In the second study, prophylaxis was used for six months and was as effective as triple antiretroviral drug regimens given to the mother as prophylaxis in reducing postnatal transmission [43]. It will be important to investigate whether prophylaxis can be extended further to cover the normal duration of breastfeeding.

### Dark clouds of deteriorating infant feeding practices

Despite these important scientific breakthroughs, there is unfortunately a dark cloud. And this dark cloud is provided by the shifts in infant feeding practices that have occurred in many places, inspired by the fear of HIV transmission. In many places, HIV-infected women in communities that previously had good uptake of full breastfeeding have shifted away from breastfeeding or have opted to wean their infants much earlier than they usually would have.

In sobering new data from rural Rakai, Uganda, mortality among infants born to HIV-infected mothers who elected to abstain from all breastfeeding was increased six-fold compared with infants born to mothers who elected to breastfeed [44]. In a clinical trial in Botswana, mortality among uninfected infants born to HIV-infected mothers was doubled among those randomized to abstain from breastfeeding compared with those randomized to short breastfeeding [45]. These two reports together suggest that the excess mortality due to replacement feeding is higher in programmatic than in clinical trial settings.

However, even in a clinical trial conducted in a country with more resources and where reasonable efforts were made to provide appropriate measures for "safe" formula feeding, a two-fold increase in uninfected child mortality was reported [45]. HIV researchers have now learned the same lessons as studies in the 1960s and 1970s that demonstrated that shifts away from breastfeeding increases infant mortality [46].

### Is "no benefit" equal to "no harm"?

In the Botswana trial, mortality among uninfected infants less than six months of age was increased about two-fold among those whose mothers were randomized to abstain from breastfeeding compared with those randomized to breastfeed. But abstinence from breastfeeding was able to reduce HIV transmission. Sadly, adding together these competing risks, there is *no net benefit *in terms of HIV-free survival of abstinence from breastfeeding [45].

**Figure 3:**
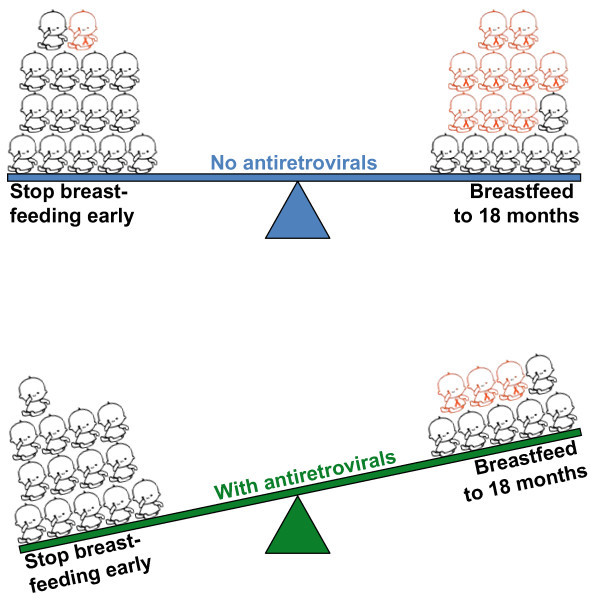
**Balancing adverse outcomes in breastfed and non-breastfed infants**. Panel A - When effective antiretroviral drugs are not provided, abstinence from breastfeeding or early weaning may result in no benefit for HIV-free survival, i.e., number of extra uninfected deaths caused = number of HIV infections prevented. Panel B - When effective antiretroviral drugs are provided, abstinence from breastfeeding or early weaning results in worse outcomes, i.e., number of extra uninfected deaths (in black outline) caused > number of HIV infections (in red outline) prevented.

We have recently completed a clinical trial in Lusaka, Zambia, which examined the period of four to 24 months in greater detail. We, like the Botswana group, also reported *no net benefit *of shortening the usual duration of breastfeeding on HIV-free survival [42]. When we investigated why we observed no benefit of early cessation of breastfeeding by examining what the women in the intervention and control groups actually did, we found that the risk of uninfected child mortality to 24 months was increased about three-fold if women stopped breastfeeding early compared with breastfeeding for 18 months or longer [47]. Stopping breastfeeding earlier than normal did reduce transmission. In other words, the benefit of HIV prevented was canceled out by the harm of uninfected child deaths caused by other infectious diseases (Figure 3).

These results are not reassuring about the safety of abstinence from breastfeeding or early weaning. To the contrary, "no benefit" does not mean the same thing as "no harm". A key point is that both of these examples are in the absence of effective antiretroviral regimens. When we provide antiretroviral drugs (using almost any of the approaches described above), postnatal transmission is substantially reduced.

Viewed in this context, the elevations in uninfected child mortality caused by abstinence from breastfeeding or caused by early weaning are no longer justified by HIV prevention efforts. The numbers of HIV infections prevented are now considerably less than the numbers of replacement feeding-related deaths caused. When antiretroviral drugs are provided, what was previously "no benefit" now becomes harm. This is not because the antiretroviral drugs are harmful, but because in the delicate risk-benefit balance, mortality caused by abstinence from breastfeeding or shortening the duration of breastfeeding is now greater than the amount of HIV prevented (Figure 3).

**Figure 4:**
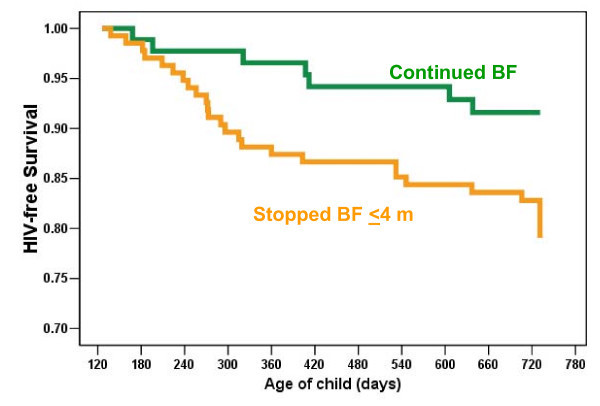
**HIV-free survival by breastfeeding practice**. HIV-free survival of children born to HIV-infected women with CD4 counts >350 is better if breastfeeding continues than if breastfeeding stops early [47].

This can be seen most clearly among women who have high CD4 counts and do not meet criteria for antiretroviral treatment. This group of women is at lower risk of transmitting even if no interventions are provided. As we observed in our cohort in the Zambia Exclusive Breastfeeding Study, for women with higher CD4 counts, there were worse infant outcomes if breastfeeding was shortened. In this group, where the HIV transmission rate is lower, the risks of prematurely truncating breastfeeding outweigh any benefits to prevent transmission [47] (Figure 4).

### Should we burn down the forest to save the trees?

In the quest for eliminating HIV transmission, we need to be cautious of the steps we take to get there. We can save the child from HIV, but have to be careful that we do not increase their risk of dying from other diseases. HIV prevention is not enough: the goal should be healthy children. The integration of adult HIV treatment with PMTCT has shown that it is possible to address both maternal health and prevent mother to child transmission. We should now turn to integrating these activities with programmes to protect child survival.

### Integrating PMTCT and child survival

The HIV-exposed uninfected child living in environments where HIV is the most prevalent is at risk for many diseases, not only HIV. We add to the jeopardy of this child by some of the interventions we provide as part of PMTCT programmes. This jeopardy is increased with a single-minded focus only on HIV prevention without consideration of broader inventions that improve infant health and development.

The unprecedented resources that have been made available to address HIV have overshadowed the resources available for routine child survival. Many simple interventions known to be of benefit to high-risk children in low-resource settings are not being implemented, perhaps because they do not have the cache and the advocates that HIV prevention has. The HIV-exposed uninfected child is also different to all other uninfected children because his or her mother is HIV positive. This has profound social ramifications of stigma, familial loss and deterioration of maternal health, all of which may affect the well-being of the uninfected children [48].

It also appears that there might be biological deficits associated with having been exposed to HIV [49]. Those who look for correlates of protective immunity in the natural context have written of the immunologic advantage among those lucky enough to have escaped HIV infection [25,26]. But there may be an immunologic disadvantage that makes exposed, uninfected infants more vulnerable to other diseases [48]. We are yet to properly understand what the long-term clinical consequences are for the hundreds of thousands of children exposed to HIV, but who survive uninfected.

## Conclusion

The PMTCT field can be credited with some of the most important breakthroughs in HIV prevention research. We have much to celebrate, but there are many challenges ahead. HIV treatment and PMTCT programmes should not be implemented as distinct and competing programmes, but can and should work hand in hand so that maternal health is addressed alongside prevention of HIV.

The first priority should be to provide access to HIV treatment for all pregnant and lactating women with CD4 counts of <350 cells/mm^3^. Every effort should be put into programmes to ensure that this happens. For children born to mothers with higher CD4 counts, there are several options, either extended maternal regimens or infant prophylaxis. Both work to prevent transmission. But this is not enough.

These programmes also need to link with child survival programmes, including high-quality counselling to support lactation, so that prevention of HIV can occur in a context that ensures the health and well-being of children.

## Competing interests

The authors declare that they have no competing interests.

## Authors' contributions

LK wrote the first draft. All other authors revised the manuscript for critical content.
